# Bacterial diversity of wild-caught *Lutzomyia longipalpis* (a vector of zoonotic visceral leishmaniasis in Brazil) under distinct physiological conditions by metagenomics analysis

**DOI:** 10.1186/s13071-017-2593-7

**Published:** 2017-12-29

**Authors:** Ana Clara Araújo Machado Pires, Luís Eduardo Martinez Villegas, Thaís Bonifácio Campolina, Alessandra Silva Orfanó, Paulo Filemon Paolucci Pimenta, Nágila Francinete Costa Secundino

**Affiliations:** 1 Laboratório de Entomologia Médica, Centro de Pesquisas René Rachou/FIOCRUZ, Av. Augusto de Lima, 1715, Belo Horizonte, MG 30190-002 Brazil; 2 Departamento de Morfologia, Universidade Federal de Minas Gerais/UFMG, Av. Pres. Antonio Carlos 6627, Belo Horizonte, MG 31270-901 Brazil

**Keywords:** Wild-caught, *Lutzomyia longipalpis*, Metagenomic analysis, Microbiota, *Leishmania*

## Abstract

**Background:**

The leishmaniases are a group of diseases caused by protozoans of the genus *Leishmania*, which are transmitted by the bite of phlebotomine sand flies. In the New World, *Lutzomyia longipalpis* is the most important vector of visceral leishmaniasis and is a proven vector for *Leishmania infantum chagasi* in Brazil. During development within the vector, *Leishmania* can interact with a variety of microorganisms such as fungi and bacteria. The presence of bacteria in the midgut of sand flies can influence the development and survival of the parasite.

**Results:**

The bacteria-targeted metagenomic analysis revealed different community compositions between the distinct physiological stages of those tested. The amplicon-oriented metagenomic profiling revealed 64 bacterial genera and 46 families. By crossing the taxa indices from each experimental condition a core composed of 6 genera was identified (*Enterobacter*, *Serratia*, *Stenotrophomonas*, *Enhydrobacter*, *Pseudomonas* and *Chryseobacterium*).

**Conclusions:**

The observed dynamic nature of the bacterial community expands the knowledge pertaining to the tripartite host-microbiota-pathogen interactions. Further studies addressing how laboratory and field collected communities differ are critical to successfully develop control strategies based on bacterial symbionts and paratransgenesis, as already tested in other arthropod vectors.

**Electronic supplementary material:**

The online version of this article (10.1186/s13071-017-2593-7) contains supplementary material, which is available to authorized users.

## Background

Leishmaniases are a group of diseases caused by protozoans of the genus *Leishmania* Ross, 1903 [1], which are transmitted by the bite of phlebotomine sand flies. The diseases present different clinical manifestations, such as visceral leishmaniasis (VL) [2]. The World Health Organization estimates that 90% of global VL cases occurred in 6 countries: Bangladesh, Brazil, Ethiopia, India, South Sudan and Sudan. In the New World, *Lutzomyia longipalpis* (Lutz & Neiva, 1912) is the most important vector of VL and is a proven vector for *Leishmania infantum chagasi* in Brazil [3].

The female sand fly can acquire *Leishmania* spp. parasites during blood feeding on an infected vertebrate. The *Leishmania* life-cycle within the insect vector is extracellular with parasite development restricted to the gut lumen [4, 5]. During development within the vector, the parasites can interact with a variety of microorganisms such as fungi and bacteria. The presence of bacteria in the midgut of sand flies can influence the development of the parasite by competing for nutrients and adhesion site in the gut of the vector, and by reducting the intestinal pH [6]. Studies have shown that microbiota from the sand fly’s midgut have an effect on *Leishmania* development and survival. Additionally, in vitro experiments have shown that *Serratia*, *Bacillus* and *Haemophilus parainfluenzae* induced lysis of promastigote forms [7–9].

The gut microbiome of the *Lu. longipalpis* colony was recently shown to be essential for survival of the parasite [10]. An antibiotic-mediated decrease in midgut microbiota impaired *L. infantum* survival in the sand fly, inhibited parasite growth, and differentiation to the infectious metacyclic form was observed [10]. Furthermore, when *Lu. longipalpis* was pre-fed with *Pseudozyma*, *Asaia* or *Ochrobactrum*, a reduced parasite survival rate was observed [11].

Microbiota can be modulated by blood meal. The number of bacteria within the midgut of *P. duboscqi* increases two days after a blood meal and decreases after blood meal digestion [12]. In addition to blood, which is required only by females for egg development, adult sand flies are also plant feeders [13]. The different food sources available during each sand fly life stage can influence the midgut microbiota. The insect is exposed to a diverse range of microbial communities during its lifespan that will contribute to form the midgut microbiota. The larval stages feed on animal feces and plant material and ingest microorganisms from the soil environment. Many of these microorganisms can be found in the gut of the adult immediately post emersion [12]. Adult sand flies have the opportunity to ingest bacteria from plants and vertebrate hosts that are used as a food source [6, 13, 14].

The composition of *Lu. longipalpis* microbiota includes bacterial taxa from the genera *Acinetobacter*, *Stenotrophomonas*, *Pseudomonas*, *Flavimonas*, *Enterobacter*, *Klebsiella*, *Bacillus*, *Staphylococcus*, *Serratia*, *Yokenella*, *Stenotrophomonas*, *Burkholderia*, *Citrobacter*, *Escherichia*, *Pantoea*, *Morganella* and *Weeksella* [15–18]. A metagenomic approach developed by McCarthy et al. [19] revealed the presence of sequences from bacteria, fungi, protist parasites, plants and metazoans in field-caught male and female *Lu. longipalpis*. Additionally, in a recent study of our group, the microbiota of field collected *Lutzomyia intermedia* was profiled by targeting 16S rRNA sequences representing midgut samples from unfed, blood-fed or gravid sand flies. The metagenomic analysis revealed that bacterial richness and abundance varied between the studied groups [20]. When considering the same model species, the community composition of the sand fly gut microbiota was modulated by the type of meal and by the presence or absence of *Leishmania* [10]. Our aim was to explore the dynamic bacterial community profiles of wild-caught *Lu. longipalpis* during distinct physiological conditions, including the ecological disturbance caused by *Leishmania* parasites upon the autochthonous adult host microbiota.

## Methods

### Parasite culture

*Leishmania infantum chagasi* (MHOM/BR/1970/BH46) was cultured in M199 medium supplemented with 10% Fetal Bovine Serum (FBS), penicillin (100 U/ml), streptomycin (50 μg/ml), glutamine (12.5 mM), HEPES (40 mM), adenine (0.1 mM) and 2.5 μg/ml hemin (Sigma-Aldrich, St. Louis, MO, USA) and kept in a BOD incubator (FANEM, model 347CD) at 26 °C in the laboratory.

### Sand fly collection and maintenance

Wild *Lu. longipalpis* sand flies were collected in the Lapinha Cave, which is a non-endemic leishmaniasis area located at Lagoa Santa, Brazil (19°3'S, 43°57'W) using CDC light traps. Unfed female sand flies were separated under stereomicroscopy and allowed to feed ad libitum on a 50% sucrose solution at 25 °C and 95% humidity. They were kept at the insectary of the Laboratory of Medical Entomology of the Centro de Pesquisas René Rachou for at least 3–4 days before the feeding experiments. The sand fly species was morphologically identified according to Young & Duncan, 1994 [21].

### Feeding and infection

Female *Lu. longipalpis* were divided into 3 groups of 100 insects each and allowed to feed on (i) sucrose (non-blood-fed, UF); (ii) uninfected blood-fed (BF); and (iii) infected blood-fed (BFI). Sand flies from the UF group were allowed to feed only on sucrose and separated for DNA extraction using (Qiagen, Hilden, Germany). The experimental infections were conducted as described [22]. Sand flies were fed through a membrane-feeding assay consisting of a glass-feeding device containing heparinized mouse blood (drawn intracardially from BALB/c mice) with heat-inactivated serum. The blood meal was offered to the BF and BFI groups, the BFI meal consisted of 4 × 10^6^ parasites/ml of *L. infantum chagasi* promastigotes. Fully engorged female *Lu. longipalpis* sand flies took a blood meal of approximately 0.2–0.3 μl each. Blood engorged flies were separated and maintained at 26 °C and 75% humidity and were provided 30% sucrose ad libitum. Three and seven days post-infection, 10 flies from the infected group were anesthetized with CO_2_ and killed in 5% soup solution. The midguts were dissected and transferred to the tubes containing 30 μl of PBS. The guts were macerated briefly using a plastic pestle, and then spun twice at 800× *rpm* for 1 min to remove the debris. A 10 μl sample of the supernatant was counted under a hemocytometer (Additional file 1: Figure S1). In addition, 30 sand flies from UF, BF and BFI groups were dissected under a stereoscope in a biosafety cabinet and separated into groups according to their physiological condition at the 3rd day after the blood feeding and stored in micro centrifuge tubes) at -20 °C for DNA extraction. Following the blood digestive process on the 7th day, 30 sand flies from BF group were separated as described above and re-grouped or re-named as gravid (GR). This group was separated according their developing ovaries and verified to confirm the physiological condition and they were stored at -20 °C for downstream DNA extraction.

### DNA extraction

Before the DNA extraction process, each pooled sample consisted of 30 sand flies from each experimental group and was surface sterilized by rinsing with distilled sterile water and then submerged for 1 min in 1% hypochlorite, washed three times with PBS [23] and dissected in a biosafety cabinet (as described above). The genomic DNA was extracted using the DNeasy Blood and Tissue Kit (Qiagen) following the manufacturer’s manual.

### Amplicon-oriented metagenomic bacterial taxa profiling

The DNA samples were carefully packed on dry ice and shipped to Macrogen, Korea. They were sequenced using the Illumina MiSeq Next-Generation Sequencing (NGS) platform targeting the 16S rRNA gene (300 bp paired end reads covering the V3-V4 hypervariable region) in accordance with Macrogen’s in-house pipelines accounting for: QC, Operational taxonomic unit (OTU) clustering at a 97% similarity threshold (CD-HIT-OTU), microbial community richness (Mothur v.1.33.0), and taxonomic assignment (Silva rRNA Database and in-house classifier software with an 80% minimum confidence value). To assess whether community homogeneity (alpha-diversity measure) was modulated by blood feeding and pathogen presence, we used the Shannon index [24] for each sample as it measures the evenness of the bacterial communities.

### Phylogenetic dendrogram visualization

The phylogenetic relationship between the genus level OTUs (g-OTUs) predicted within the four tested groups was explored for illustrative means using NCBI’s taxonomy browser (http://www.ncbi.nlm.nih.gov/Taxonomy/CommonTree/wwwcmt.cgi). The generated Philip format tree was visualized and edited using the Interactive Tree Of Life (iTOL) web-based tool [25].

### Microbial ecology multivariate analyses

Comparative community diversity analyses were performed on the bacterial abundance data matrix to evaluate a potential biological pattern or signature profiles discriminating the compared sand fly groups. The ecological community comparisons, based on β-diversity dissimilarity measurements, were performed on a matrix encompassing the bacterial OTU relative abundance data pertaining to the four tested conditions that were previously explained. Sequencing methods (considering elements such as the platform and primers), data QC processing, and the taxonomic profiling database used (among other factors) may bias community composition analyses, particularly if working at the species and genus ranks [26–29]. Thus, in order to perform more robust analyses by reducing the number of zeroes in the data matrix we opted to work at the taxonomic family level for a more conservative approach encompassing OTU counts at a higher rank. The variation in the community structure was explored by multivariate analyses based on pair wise distances between the four tested groups. A variance stabilizing transformation was applied to the relative abundance matrix to reduce the dispersion expressing the values as arcsin√x (see Ramette [30], Mason et al. [31]). Exploratory multivariate analyses were performed on the transformed data set of the bacterial abundance profiles following suggested microbial ecology parameters [30, 32–34]. A hierarchical clustering and non-metric multidimensional scaling (NMDS) analyses of the Bray-Curtis (B-C) similarity index were generated using R-studio v.0.98.507. To further interpret the biological patterns and clustering of the experimental groups, we searched for the f-OTU correspondence (based on their relative abundance) with the biological conditions that each group represents. A detrended canonical correspondence analysis (DCA) was conducted to determine if a linear or unimodal ordination method could be applied to the data. The longest gradient obtained was larger than 3.0 SD units, which indicated that a unimodal model-based constrained method is a suitable test [30, 35]. Thus, a canonical correspondence analysis (CCA) was performed using an additional constraining explanatory matrix with “dummy” binary variables and referring to the following biological conditions: blood present in the midgut, infection with *Leishmania* parasites, and previous exposure to blood (to consider the case of gravid sand flies). By constraining the analysis, not only did we test for correspondence (or ecological preference) [30] between f-OTUs and the food source based groups, but also explored how the explanatory variables we proposed could influence the variance within the abundance matrix and the localization of the objects in the ordination space.

## Results

### Composition of the bacterial community associated with wild-caught *Lu. longipalpis*

The bacteria-targeted metagenomic analysis revealed different community compositions between the distinct physiological stages of those tested. Sequencing revealed a total of 1,178,018 high quality reads, which were grouped as follows: UF, 93,244; GR, 284,017; BF, 388,670; and BFI, 412,087. The amplicon oriented metagenomic profiling revealed five phyla (Actinobacteria, Bacteroidetes, Firmicutes, Proteobacteria and Spirochaetes), 64 bacterial genera and 46 families. When considering the predicted g-OTUs for each of the experimental conditions, the UF group harbored the highest number of OTUs with 57 genera and 14 exclusive (*Zymobacter*, *Aquabacterium*, *Empedobacter*, *Myroide*, *Petrimonas*, *Sphingobacterium*, *Niabella*, “*Candidatus* Cloacimonas”, *Sporolactobacillus*, *Lactobacillus*, *Streptomyces*, *Lentzea*, “*Candidatus* Lumbricincola” and *Edwardsiella*); followed by the GR group with 46 genera and 3 exclusive (*Acidovorax*, *Plesiomonas* and *Sphaerotilus*), BFI (the infected group) with 22 genera, 3 exclusive (*Rickettsiella*, *Mucispirillum* and “*Candidatus* Cardinium”), and finally, the BF group with 6 genera and none exclusive. Crossing the taxa indices from each experimental condition, a core composed of 6 genera was identified (*Enterobacter*, *Serratia*, *Stenotrophomonas*, *Enhydrobacter*, *Pseudomonas* and *Chryseobacterium*).

**Fig. 1:**
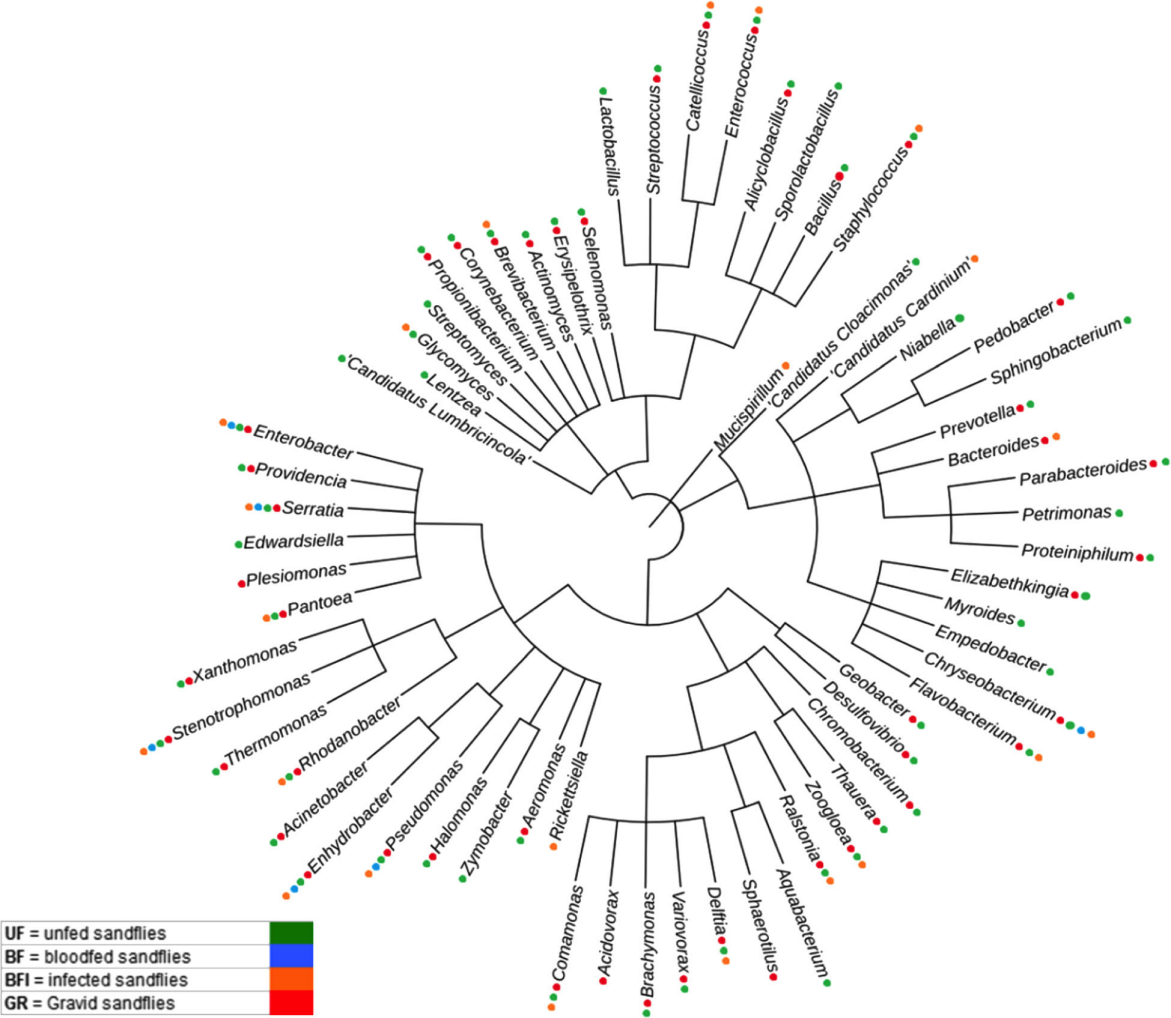
Phylogenetic dendrogram of bacterial genera identified in each of the experimental conditions of the wild-caught *Lu. longipalpis*. The identified g-OTUs are shown followed by a color key that represents the physiological conditions of the sand fly females as described in Methods: UF (*green*); BF (*blue*); BFI (*orange*); and GR (*red*)

In terms of the community homogeneity among the bacterial taxa, each of the groups presented the following Shannon indices: UF, 1.42; GR, 1.12; BF, 0.70; and BFI, 0.48. The color code used to show the presence (or absence) of the g-OTUs in each group (Fig. 1) led us to seek a potential biological pattern.

### Comparisons of community richness and abundance

The bacterial f-OTU richness and abundance associated with wild-caught *Lu. longipalpis* varied between the studied groups. In the UF and GR groups, the family *Pseudomonadaceae* was the most prevalent and accounted for 76,031 and 204,688 HQ reads (81.60 and 72.23%, respectively). *Enterobacteriaceae* was the most abundant family in the BF and BFI groups and represented 236,442 and 393,927 HQ reads of each group (60.83 and 95.60%), respectively.

After the blood meal, bacterial abundance in terms of the raw reads increased when compared to the UF and GR groups. This was concomitant with the loss of f-OTU richness. Blood presence in the midgut negatively modulated the abundance of *Pseudomonadaceae*, *Moraxellaceae* and *Flavobacteriaceae* in the BF group, and *Enterobacteriaceae* and *Xanthomonadaceae* were the dominant taxa. In the BFI group, the abundance of *Enterobacteriaceae* increased drastically when compared with the rest of the groups (Additional file 2: Table S1).

### Multivariate analyses exploring the underlying bacterial profiles

Initially, exploratory unconstrained methods were applied to explore how the bacterial abundance profiles (at the family taxonomic rank) may act as a “signature” capable of discriminating among the experimental groups under distinct physiological conditions and based on the influence of the inner ecosystem in which they develop. Both the hierarchical clustering and NMDS revealed two biologically relevant patterns based on a B-C distance matrix (Additional file 3: Figure S2). First, a clear resemblance between the bacterial abundance profiles of the sand flies from the GR and UF groups separated them from the two blood-fed groups (the BF and BFI groups). Secondly, the presence of *Leishmania* parasites may modulate the bacterial community composition (diversity and abundance-wise) because the sand flies from the BFI group clustered separately from its BF counterpart group.

**Fig. 2:**
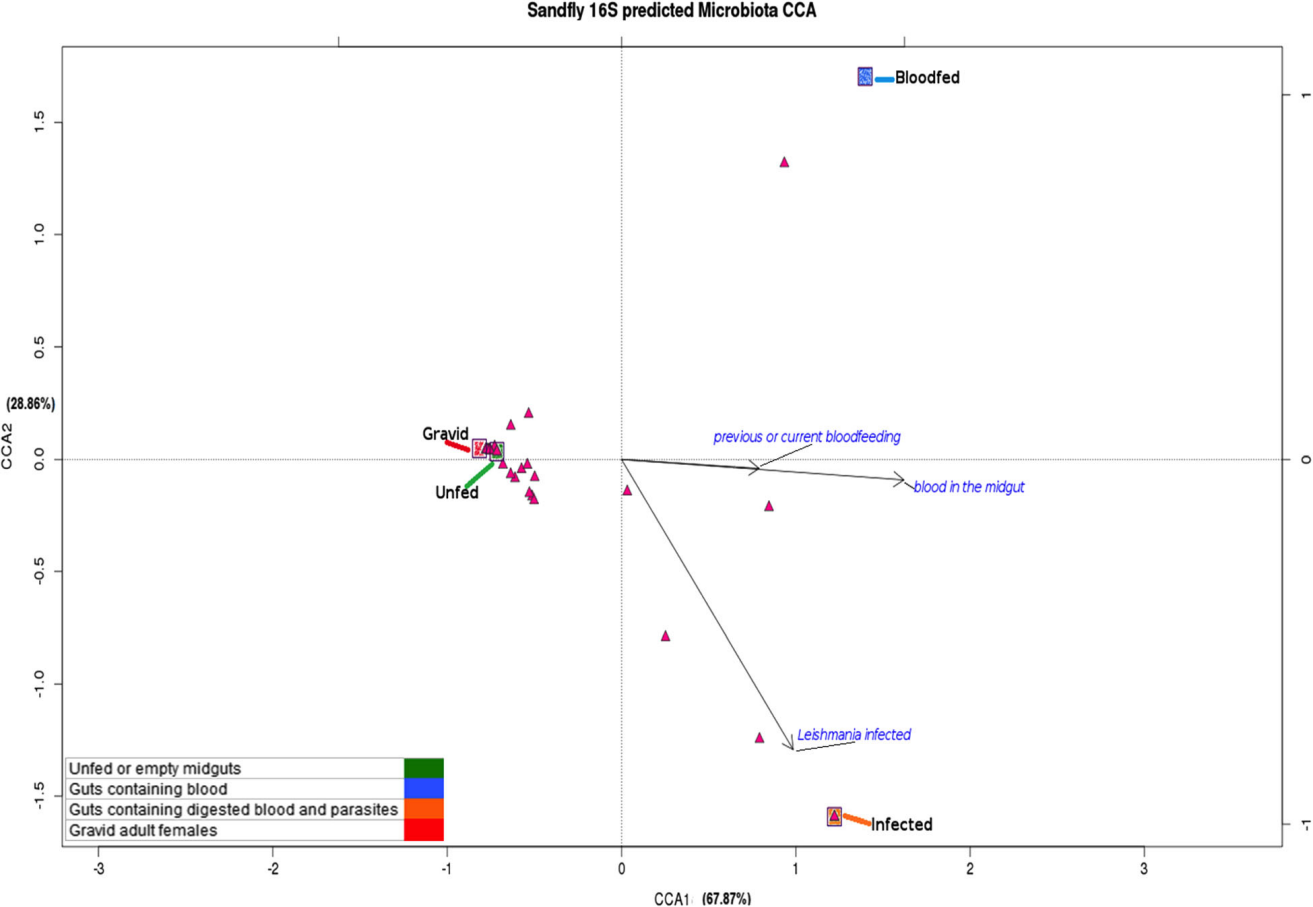
Constrained multivariate analysis on bacterial abundance profiles of different wild-caught *Lu. longipalpis* groups based on their food source. CCA (scaling 2) showing the grouping of the experimental sand fly groups according to their f-OTU abundance profiles. The constrained CCA1 and CCA2 axes explain approximately 97% of the variance within the variance matrix of bacterial abundance. Both UF and GR groups co-localized upon the upper left quadrant and separated along CCA1 from the two blood-fed groups (BF and BFI groups). The presence of the parasite draws the BFI group away from its BF counter-group along CCA2. The length of the vectors in blue corresponds to the importance that each of the explanatory (constraining) variables had upon the clustering pattern. Each of the f-OTUs (*red*) is distributed in the ordination space and its preference for an “ecological niche” can be attributed to its proximity to each of the experimental groups

Based on the exploratory unconstrained methods, a CCA (scaling 2: focusing on response variables) was executed following the recommendations found in the microbial ecology literature. This technique not only seeks a biological pattern driving the spatial distribution of the objects influenced by the OTU abundance profiles, but also inquires how each OTU responds to the explanatory variables. The CCA analysis coupled to the explanatory variables allowed us to obtain an estimate of the proportion of variance explained by each axis (CCA1 = 67.87% and CCA2 = 28.86%, Fig. 2). Thus, encompassed in these two constraining axes, we found 96.73% of the total variance within the dissimilarity matrix. As observed in the NMDS plot (and the hierarchical cluster plotted on top), a biological pattern based on a food source modulating the bacterial profile can be elucidated. Along the CCA1 axis, the UF and GR groups co-localize and separate from both blood-fed groups (BF and BFI groups). Along CCA2, we observed how the presence of the pathogen modulates the bacterial profile and draws this group apart from its counterpart and toward the lower right quadrant of the ordinate space (Fig. 2). By constraining the analysis, we were also capable of assessing the impact of the explanatory variables. The blue vector arrows shown in the lower right quadrant clearly indicate the influence of blood as a food source and infection as driving forces clustering the experimental groups (the length of the vector is indicative of its effect upon the spatial distribution of the elements).

The other layer of information obtained from this method is the ecological preference towards each of the nutritional/physiological states that each f-OTU showed and their response to the explanatory vectors. This is assessed by: their location within the ordination space and their proximity to each of their “ecological niches” or the sand fly group and a right-angled projection onto the vector arrows. The ecological correspondence is as follows: BF group = *Xanthomonadaceae*; BFI group = *Enterobacteriaceae*, *Enterococcaceae*, *Bacteroidaceae*, *Coxiellaceae*, *Flameovirgaceae*, *Deferribacteraceae*, *Glycomycetaceae*; and “co-localized” UF and GR groups = *Streptococcaceae*, *Bacillaceae*, *Thermotogaceae*, *Actinomycetaceae*, *Planococcaceae*, *Neisseriaceae*, *Veillonellaceae*, *Pseudomonadaceae*, *Burkholderiaceae*, *Aeromonadaceae*, *Staphylococcaceae*, *Corynebacteriaceae*, *Erysipelotrichaceae*, *Sinobacteraceae*, *Lactobacillaceae*, *Mycoplasmataceae*, *Spirochaetaceae*, *Geobacteraceae*, *Halomonadaceae*, *Rikenellaceae*, *Propionibacteriaceae*, *Alicyclobacillaceae*, *Desulfovibrionaceae*, *Brevibacteriaceae*, *Chromatiaceae*, *Rhodocyclaceae*, *Moraxellaceae*, *Porphyromonadaceae*, *Prevotellaceae*, *Xanthomonadaceae*, *Streptomycetaceae*, *Actinosynnemataceae*, *Comamonadaceae*, *Cytophagaceae*, *Flavobacteriaceae*, *Sphingobacteriaceae* and *Sporolactobacillaceae.*

## Discussion

Most studies evaluating the microbiota of phlebotomines have used classical microbiological techniques such as bacterial culture and isolation, which are limited in their ability to broadly profile the insect’s microbiota since most bacteria have not been cultured to this date [36]. Our study analyzed the microbiota composition of wild-caught *Lu. longipalpis* from Lapinha Cave by 16S rRNA gene-oriented metagenomics through a technique that allows for the detection of a large number of bacterial taxa for which culture is not yet feasible.

Meta-taxonomic profiling revealed 64 unique bacterial OTUs at the genera level associated with the *Lu. longipalpis* groups. The majority of these genera include bacteria commonly associated with soil, water and plants, which can be acquired during the feeding habits of sand flies, i.e. soil during the larval stage and the sap of plants during the adult phase [9, 12, 14, 37]. In terms of g-OTU numbers, the group with the highest diversity was the UF group (57 genera), followed by GR (46 genera), BFI (22 genera) and BF (6 genera). When addressing community richness in terms of evenness, the measures of Shannon indices revealed that bacterial OTUs were more diverse and equitably distributed in gravid and sugar-fed sand flies, whereas the read abundances were less homogenously distributed among the dominant taxa in the community when exposed to blood and the invading pathogen.

Our community composition study can be contrasted with a recent study that also targeted bacterial profiling of sand fly microbiota in different physiological conditions [10, 20]. A metagenomic study by Kelly et al. [10] also targeted *Lu. longipalpis* microbiota by taking into account the modulation effect of pathogen infection as we did. In their study, *Acetobacteraceae* and *Enterobacteriacae* were the most abundant families in the blood-fed and infected groups and *Methylobacteriaceae*, *Microbacteriaceae*, *Shingomonadaceae*, *Alcaligenaceae* and *Brucellaceae* associated with their tests groups. We also detected the predominance of *Enterobacteriacae* in the same conditions. In contrast, we did not detect *Acetobacteraceae* in those groups and *Xanthomonadaceae* were the co-dominant f-OTU in the blood-fed group. These diversity divergences for the same host species are plausible and potentially because wild-caught and laboratory colonized *Lu. longipalpis* harbor different bacterial consortia both in terms of abundance and richness. Such a phenomenon has been observed before in other insects, such as *Anopheles* [38]. In a recent study, Fraihi et al. [18] demonstrated that microbiota composition shows diversity decline at the species level during the time course of the transmission period. We performed experimental infection with sand flies from a non- endemic area and therefore we did not analyze the transmission period. However our BFI group also showed a decrease on the microbiota composition when compared with the UF and GR groups. Together, these data suggest that successful invasion and concomitant transmission of these vector-borne parasites is reflected by the ecological disturbance of the native bacterial community in the host.

Notwithstanding the shifting bacterial diversity profiles, a group of permanently associated taxa was present, in agreement with reports for other vectors. This core set of bacterial OTUs (mostly at the genus and family taxonomic level) remains present in the internal ecosystem through different life stages and between physiological conditions and in multiple body compartments. Inner vector biology effectors such as nutrition, immune response and age would then modulate this core group in terms of relative abundance [39–42]. Their importance would lie in their potential role as providers of nutrients, gene functions in key host-symbiont metabolic networks [43–46], or their involvement in hematophagy related redox homeostasis [47].

Among the core taxa identified, the genera *Enterobacter* and *Serratia* (*Enterobacteriaceae* family) have been previously described in Old and New World sand flies including in *Lu. longipalpis* from Lapinha Cave [6, 10, 12, 15–18, 20, 37, 48–50]. This f-OTU is commonly reported as an abundant component of the bacterial consortium in hematophagous arthropods and thrives particularly in the midgut of blood-fed and *Plasmodium*-infected anophelines [38, 39] and in other adult female culicids [51]. Interestingly, in *Aedes aegypti*, taxa from this family significantly thrive more in sugar-fed mosquitoes than blood-fed mosquitoes [41] in which *Comamonadaceae* [46] and *Pseudomonadaceae* [41] dominantly proliferate when exposed to blood. In our study, *Enterobacteriaceae* was the most abundant in both the BF and BFI groups.

At the genus level, *Stenotrophomonas* was the most prevalent in the BF group. This g-OTU was previously described in wild [15, 17, 18] and blood-fed female *Lu. longipalpis* [10, 15], as well as in *P. duboscqi* [12]. As observed in *Anopheles gambiae* [39], an increase of bacterial biomass (in terms of number of reads) in blood-fed individuals was due to the higher abundance of *Enterobacteriaceae*, which occurs along with a reduction in the OTU numbers. This ecological unevenness is reflected concordantly in the trend of the Shannon indices estimated for the sand fly groups exposed to blood feeding and is more acute when infective feeding occurred.

The genus *Pseudomonas* was the most prevalent in the UF and GR groups and accounted for 76,031 and 204,688 HQ reads. This g-OTU was previously identified in *Lu. longipalpis* [10, 15, 16, 20, 37, 52] *P. argentipes*, *P. duboscqi*, *P. papatasi*, *P. perfiliewi* and *P. sergenti* [6, 12, 14, 17, 48, 53]. This is the first account of *Enhydrobacter* and *Chryseobacterium* in sand flies, genera belonging to the *Moraxellaceae* and *Flavobacteriaceae* families, respectively, whose presence in sand flies was reported recently [10]. Both genera were more abundant in the UF and GR groups than in BF and BFI groups.

By exploring the beta diversity both by a ranking method (NMDS) and a constraining method (CCA), we observed that the community composition within the sand fly holobiont is modulated by the type of meal, the presence or absence of *Leishmania* parasites, and vitellogenesis. The CCA analysis revealed a resemblance between the bacterial abundance profiles of the UF and GR sand fly groups. They both localize close along the first axis (Fig. 2) that separated them from the two blood-present midgut groups (BF and BFI). The biological effect of blood nutrition and infection, represented by the constraining vectors, clearly drive these two sand fly groups to localize separately along the CCA2 axis. Modulation of bacterial profiles by infection of a protozoa pathogen, which is reflected by constrained ordination space, had been already reported for the *A. gambiae*-*Plasmodium falciparum* model using the RDA method [39]. Recent advances in the area of microbial ecology indicated the need to study microbiomes under the light of community ecology.

The bacterial f-OTU profile of gravid females resembled that of the UF group according to their co-localization on the CCA plot. This diversity based signature would imply that the microbial assembly within the insect shows “composition resilience” when addressing the feeding behavior from the perspective of disturbance ecology tenants [54]. In this case, the bacterial community would reacquire the carbohydrate nutrition signature profile (UF) after the blood-feeding disturbance. The CCA analysis, a method based on the ecological niche theory [30, 34], allowed us to comprehend the preference that f-OTUs exhibit and thrives under particular biological conditions. Thus, their response to biotic and abiotic effectors reveals signature profiles that may aid to further elucidate the metabolic interactions between host and microbiota as we approach them as a “macrobial unit” [55] as interestingly proposed when studying holobiont phylosimbiosis [55].

## Conclusions

We described the native microbiota of wild-caught *Lu. longipalpis* under distinct physiological conditions including a pathogen infected group. The observed dynamic nature of the bacterial community expands the knowledge pertaining to the tripartite host-microbiota-pathogen interactions. It is important to further study how laboratory and field-collected communities differ in successfully developing control strategies based on bacterial symbionts so that it can be tested for other arthropod vectors.

## Additional files

Additional file 1: Figure S1. Parasite numbers in infected *L. longipalpis* (3 to 7 days after the infective blood meal). Flies were infected by membrane feeding; the midguts were dissected on the indicated days post-feeding. The parasite densities varied according to the post-infection day showing a median of 18,450 parasites on the 3rd day and 9650 on the 7th day. (PNG 743 kb)

Additional file 2: Table S1. Taxonomic profile and relative abundance of f-OTUs. Taxonomic profile and relative abundance of f-OTUs associated with different *Lu. longipalpis* experimental conditions (UF = unfed; GR = gravid; BF = blood-fed; and BFI = blood-fed infected). (PNG 1141 kb)

Additional file 3: Figure S2. Unconstrained multivariate analyses of biological patterns. NMDS plot and hierarchical clustering dendrogram showing how bacterial abundance signatures at the f-OTU level potentially discriminate between the four experimental groups. As observed across NMDS dimensions 1 and 2, sand flies from the GR and UF groups present a similar bacterial abundance profiles, whereas the blood meal seems to generate two distinct signatures that explain the spatial clustering and branching patterns along both dimensions. The NMDS analysis was plotted based on the results obtained when the stress of the meta MDS function approximated the zero value after 20 runs. (PNG 1454 kb)

## Abbreviations

BF: Uninfected blood-fed
BFI: Infected blood-fed
CCA: Canonical correspondence analysis
DCA: Detrended canonical correspondence analysis
f-OTUs: Family level operational taxonomic units
g-OTUs: Genus level operational taxonomic units
GR: Gravid
ITOL: Interactive Tree Of Life
NGS: Next-generation sequencing
NMDS: Non-metric multi-dimensional scaling
OUT: Operational taxonomic unit
UF: Non-blood-fed
