# Supplementary figures and images for: Bacterial diversity of wild-caught Lutzomyia longipalpis (a vector of zoonotic visceral leishmaniasis in Brazil) under distinct physiological conditions by metagenomics analysis
Source: Parasit Vectors. 2017 Dec 29;10:627. doi: 10.1186/s13071-017-2593-7 (PMC5747039; doi:10.1186/s13071-017-2593-7)

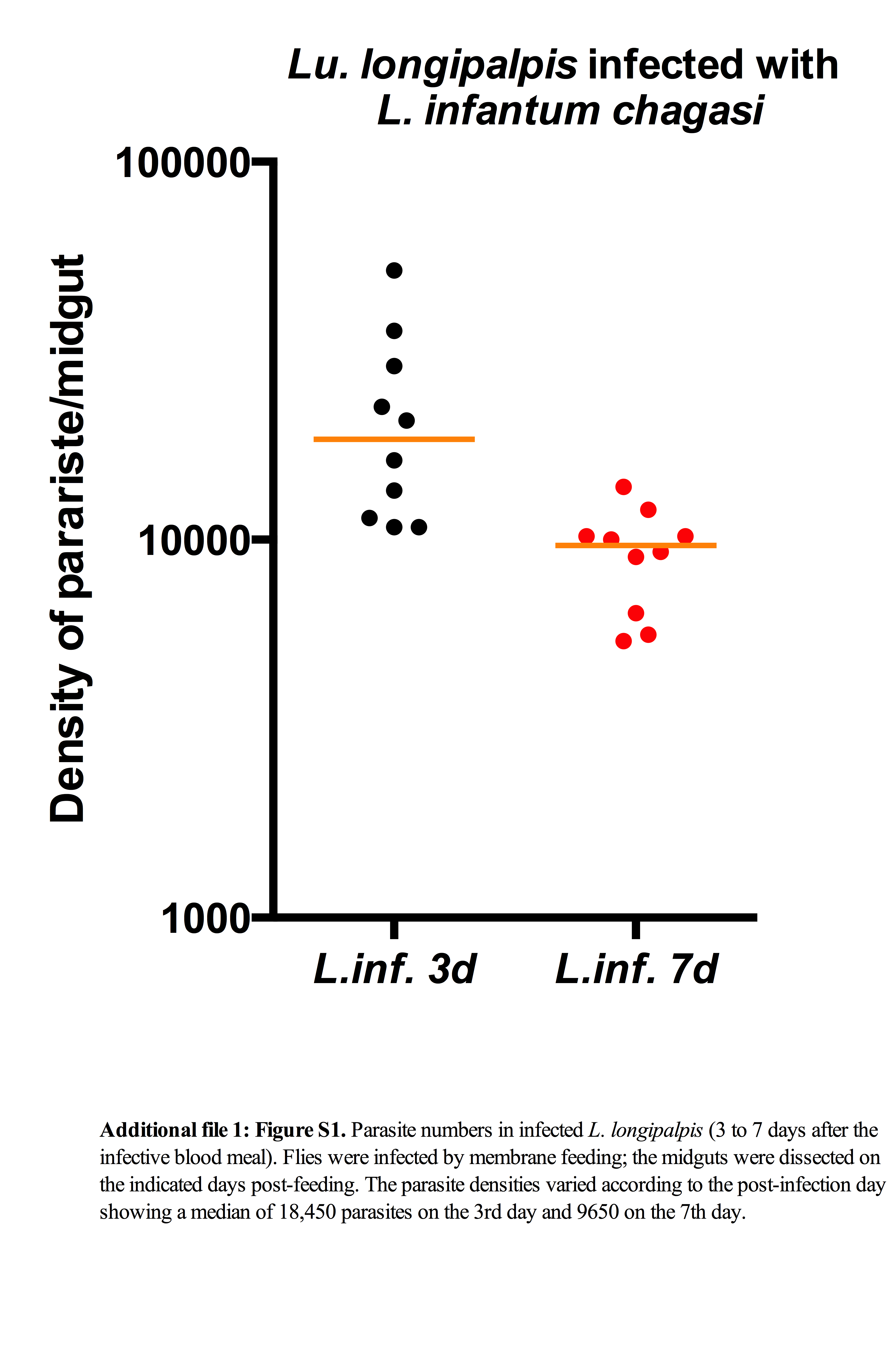

Supplement: Supplementary file 1 — Parasite numbers in infected L. longipalpis (3 to 7 days after the infective blood meal). Flies were infected by membrane feeding; the midguts were dissected on the indicated days post-feeding. The parasite densities varied according to the post-infection day showing a median of 18,450 parasites on the 3rd day and 9650 on the 7th day. (PNG 743 kb) [file 13071_2017_2593_MOESM1_ESM.png]

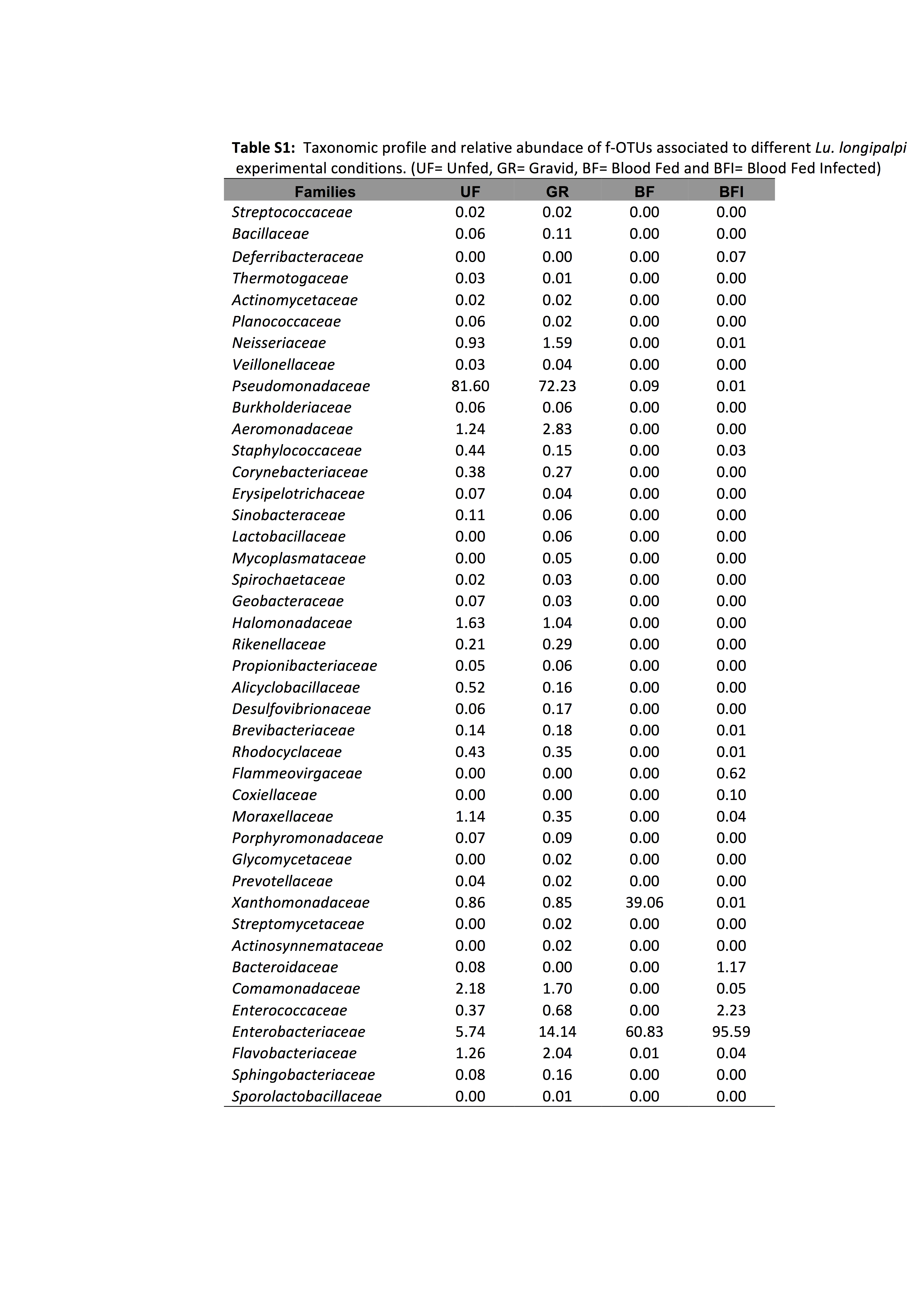

Supplement: Supplementary file 2 — Taxonomic profile and relative abundance of f-OTUs. Taxonomic profile and relative abundance of f-OTUs associated with different Lu. longipalpis experimental conditions (UF = unfed; GR = gravid; BF = blood-fed; and BFI = blood-fed infected). (PNG 1141 kb) [file 13071_2017_2593_MOESM2_ESM.png]

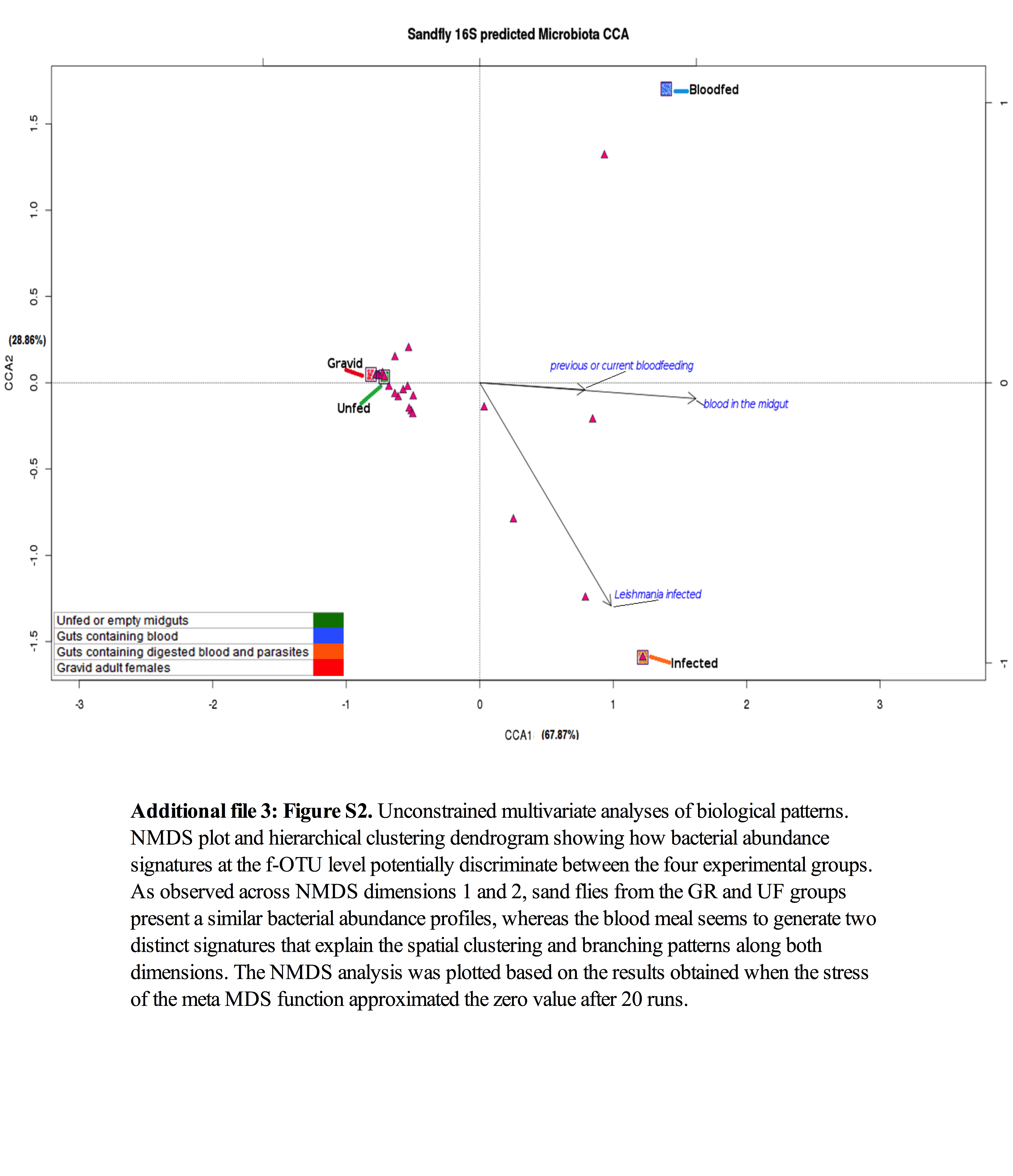

Supplement: Supplementary file 3 — Unconstrained multivariate analyses of biological patterns. NMDS plot and hierarchical clustering dendrogram showing how bacterial abundance signatures at the f-OTU level potentially discriminate between the four experimental groups. As observed across NMDS dimensions 1 and 2, sand flies from the GR and UF groups present a similar bacterial abundance profiles, whereas the blood meal seems to generate two distinct signatures that explain the spatial clustering and branching patterns along both dimensions. The NMDS analysis was plotted based on the results obtained when the stress of the meta MDS function approximated the zero value after 20 runs. (PNG 1454 kb) [file 13071_2017_2593_MOESM3_ESM.png]
